# The Prognosis of Granulomatosis With Polyangiitis: The Risk of Relapse and Mortality Based on Baseline Clinical Manifestations, Laboratory Findings, and Disease Severity: A Retrospective Cohort Study

**DOI:** 10.1002/hsr2.71239

**Published:** 2025-09-14

**Authors:** Farzaneh Kianifar, Mohammad Eslami, Soheil Tavakolpour, Noushin Afshar Moghaddam, Samira Alesaeidi, Seyed Behnam Jazayeri

**Affiliations:** ^1^ Internal Medicine Ward, Rasool Akram Hospital Center Iran University of Medical Sciences Tehran Iran; ^2^ Department of Pathology, Emam Hossein Educational Hospital Shahid Beheshti University of Medical Sciences Tehran Iran; ^3^ Dana‐Farber Cancer Institute Harvard Medical School Boston Massachusetts USA; ^4^ Rheumatology and Internal Medicine, Amir‐Alam Hospital Tehran University of Medical Sciences Tehran Iran

**Keywords:** anti‐neutrophil cytoplasmic antibody‐associated vasculitis, granulomatosis with polyangiitis, mortality, prognosis, recurrence, signs and symptoms

## Abstract

**Background and Aims:**

Granulomatosis with polyangiitis (GPA) is a highly relapsing disease with risk of mortality. The aim of the current study is to determine the relationship between baseline demographic characteristics, clinical manifestations, laboratory findings, and disease severity with patients' risk of relapse and mortality.

**Methods:**

A retrospective cohort study was performed in GPA patients diagnosed with American College of Rheumatology 1990 criteria who were referred to a tertiary care center between 2012 and April 2020. Birmingham vasculitis activity score (BVAS), clinical features, involved organs, laboratory findings, induction treatment and demographic characteristics of patients were recorded at the onset of diagnosis. Cox proportional hazards model was used to determine the factors affecting patient mortality. The predictors of relapse were analyzed using cumulative incidence function in a competing risk setting (with mortality without relapse as a competing event).

**Results:**

The cohort included 147 patients (54% male, median age: 45 [IQR: 34–61]) with a median follow‐up time of 18 months (IQR: 6–38). Overall, 55 patients died during the follow‐up leading to a survival rate of 62.5% and 179 relapses occurred among 117 patients (mean number of relapse per patient = 1.53). Independent factors for relapse included: Age > 55 (relative risk (RR): 2.50; 95% CI [1.20–2.51]), male gender (RR: 2.17; 95% CI [1.10–4.28]), rise of creatinine at baseline (RR: 2.24; 95% CI [1.15–4.35]) and nervous system involvement (RR: 2.18; 95% CI [1.21–3.96]). Significant factors for mortality were age ≥ 55 years (hazard ratio (HR): 2.40; 95% CI[1.24–4.61]), male gender (HR: 2.23; 95% CI[1.17–4.25]) rise of creatinine over 1.3 mg/dL (HR: 2.48; 95% CI [1.22–5.05), and nervous system involvement at baseline (HR: 2.16; 95% CI [1.20–3.89).

**Conclusions:**

Older age, male gender, nervous system involvement at onset and rise of creatinine are for factors that predict higher risks of relapse and mortality in patients with GPA. More intensive treatment is recommended in these subgroups of patients.

## Summary

- Male patients ≥ 55 years with nervous system involvement or rise of creatinine are at higher risk of relapse and mortality.
- More intensive treatment is recommended in these subgroups of patients.

## 1 Introduction

Granulomatosis with polyangiitis (GPA), formerly known as Wegener's granulomatosis, is a form of small to medium‐sized necrotizing vasculitis that is associated with anti‐neutrophilic cytoplasmic antibodies (ANCA) and can be life‐threatening [1, 2]. In fact, it is the most common life‐threatening small‐vessel vasculitis disease in adults. GPA has a multisystem autoimmune nature; therefore, patients may present various manifestations occupying a wide continuum at the initial evaluation and throughout the follow‐up. Ear, nose, and throat (ENT) involvements are the most frequent symptoms of GPA and are reported in 88%–100% of cases [3]. Otorhinolaryngological abnormalities are the main cause of seeking medical care in patients and include but are not limited to nasal and paranasal sinusitis, recurrent epistaxis, and otitis media [4]. Pulmonary symptoms such as chronic cough, pulmonary nodule/cavity, and pulmonary infiltration are also common [5]. Diffuse alveolar hemorrhage is a critical complication of pulmonary involvement of GPA that is life‐threatening and requires urgent management [6]. Renal involvement of GPA includes necrotizing glomerulonephritis leading to hematuria with or without RBC casts, proteinuria, rise in creatinine, and if left untreated, renal failure [7]. Mucosal/eye involvement and nervous system involvement are also prevalent with 20%–50% of patients present with such symptoms [8]. Other infrequent symptoms, such as cardiovascular [9], cutaneous [10], and gastrointestinal symptoms [11] could also be observed in GPA patients.

GPA is a highly relapsing disease with high morbidity and mortality. Involvement of various organs of the body at diagnosis such as involvement of the cardiovascular system [12], and upper airways [13], higher disease severity score based on BVAS score [14, 15], laboratory findings such as high ANCA titer and specific type of PR3 [16, 17, 18, 19], elevated inflammatory markers [20, 21], decreased hemoglobin and rise of creatinine [13], and some demographic characteristics of patients, including age and gender [22, 23], have all been suggested in the literature as indicators related to the prognosis of AAVs. However, only a few large‐scale studies included only GPA population due to the rarity of the disease [2]. Identifying the GPA patients who have a high risk of relapse or mortality can be important for prompt and more aggressive treatment with closer follow‐up of these patients. The aim of the current study is to determine the relationship between demographic characteristics, clinical manifestations, laboratory findings, and disease severity at the time of diagnosis with patients' risk of relapse and mortality.

## 2 Materials and Methods

This study is performed based on recommendations set forth by the Strengthening the Reporting of Observational Studies in Epidemiology Statement [24]. All authors have read and approved the final version of the manuscript S.B.J. had full access to all of the data in this study and takes complete responsibility for the integrity of the data and the accuracy of the data analysis. The study obtained approval from the Ethics Advisory Committee of the Research Vice‐Chancellor at Tehran University of Medical Sciences in Iran (Approval ID: IR.TUMS.AMIRALAM.REC.1399.036) before its commencement. The research was conducted in accordance with good clinical practice guidelines, and all participants provided written informed consent to participate in the study.

### 2.1 Eligibility Criteria

We included all consecutive adult (≥ 16 years) GPA patients with a Birmingham Vasculitis Activity Score for GPA (BVAS/GPA) score of at least 3, who had received either rituximab or cyclophosphamide (with or without glucocorticoids) for remission induction between January 2012 and April 2020. All patients fulfilled the 1990 American College of Rheumatology 1990 criteria [23] and/or revised Chapel Hill Consensus Conference nomenclature for GPA [25]. Only patients with documented clinical findings since diagnosis and either a minimum follow‐up of 6 months or death within 6 months of enrollment were included. Exclusion criteria concurrent treatment involving plasma exchange, methotrexate (MTX), mycophenolate mofetil, or azathioprine (AZA) as part of the induction regimen. Additionally, patients with a diagnosed autoimmune disorder, active malignancy, or a history of malignant disease within the past 3 years were excluded.

### 2.2 Data Collection

Patients' demographic features, clinical manifestations, laboratory and pathological findings, treatment regimens, and follow‐up results were recorded in a repository at each clinical visit. To systematically record the clinical symptoms of patients we used BVAS/GPA [26]. Based on this criterion, patients' clinical manifestations are categorized into nine organ systems. BVAS/GPA contains 34 predefined clinical signs/symptoms for patients that fall into two categories minor (19 items) or major (15 items). In addition, a part under “other sign/symptoms” exists that can be added to the original form by the physicians to ensure capturing most GPA‐related presentations. The severity of active disease at the time of diagnosis was quantified using the BVAS version.3 score [27]. The laboratory data included complete blood count, serum creatinine (Cr), urine analysis, immunological markers (MPO and PR3‐ANCA) inflammatory markers (Erythrocyte sedimentation rate [ESR], and C‐reactive protein [CRP]). ANCA was detected by indirect immunofluorescence and specificity for PR3‐ANCA or MPO‐ANCA was determined by enzyme‐linked immunosorbent assay. The histopathological examination was considered positive when findings were compatible with vasculitis with granulomatous changes, necrotizing vasculitis, or necrotizing glomerulonephritis. Treatment regimens included both induction and maintenance therapies. Finally, for deceased patients, the time and cause of mortality were recorded on death certificates.

### 2.3 Treatment Protocol

The treatment regimen involved rituximab (RTX) or cyclophosphamide (CYC) for induction therapy, followed by RTX, AZA, or MTX for maintenance. RTX was administered as a 1000 mg intravenous (IV) dose on Days 1 and 15 to achieve remission, with subsequent 500 mg IV infusions every 6 months for maintenance. CYC was given as 15 mg/kg pulses every 2–3 weeks during induction, followed by either MTX (15 mg/week) or AZA (2.5 mg/kg) for continued therapy. Corticosteroid use was consistent across treatment groups. All patients initially received 1–3 pulses (1000 mg) of methylprednisolone, followed by oral prednisolone (1 mg/kg/day, up to 80 mg/day). Prednisolone was gradually tapered over 6 months to a dose of less than 10 mg/day.

### 2.4 Criteria for Therapeutic Response

To evaluate the therapeutic response, the disease status of the individuals was used during follow‐up visits. Disease status was determined based on changes in patients' BVAS/GPA score. Relapse was defined as a new, worsening of, or recurrence of any item on the BVAS/GPA that required a change in therapy.

### 2.5 Statistical Analysis

This study conforms to standard guidelines of statistics [28]. All statistical analyses are performed in R software version 4.4.3 (Vienna, Austria) using packages of “cmprsk,” “survival,” “survminer,” “dplyr,” “car,” “survout” and “gmodels.” We performed a Cox proportional hazards model with calculation of hazard ratios and their 95% confidence interval for mortality. Demographic characteristics, treatments and other variables to be assessed as potential predictors of mortality in patients with AAV were determined before the study, based on a literature review. A purposeful variable selection method was used to identify predictors for inclusion in Cox model. Our purposeful selection process began by selecting any univariate variables that occurred in at least 5% of the survivor population and with a *p* value of < 0.05 for multiple analyses, after checking for multicollinearity. Multicollinearity between predictor variables was assessed using correlation analysis. Correlation coefficients > 0.7 between variables were considered to indicate potential multicollinearity; in such cases, one of the highly correlated variables was removed from the multiple regression analysis based on clinical relevance. Variables were then removed from the multiple regression model if they were nonsignificant (*p* > 0.05) and a nonconfounder (there was a < 20% change in remaining parameters compared with the full model). Age at diagnosis, disease severity, and gender were forced into the final model, as we hypothesized that these would be clinically relevant variables. The final model was fitted with statistically significant variables, confounding variables, and clinically significant variables. Kaplan‐Meier curves are presented to display the time‐to‐death data among factors that were found significant in the final Cox model and a log rank test was performed to compare survival between groups. Patients were censored if they had not reached either outcome at their last follow‐up. Patients who died without evidence of disease activity were censored on the dates of their deaths.

Regarding factors affecting relapse, we used cumulative incidence curves in a competing risk setting (with mortality without relapse as a competing event) to calculate the probability of relapse [29]. Gray's test for equivalence cumulative incidence function (CIF) was used to detect differences across groups. Direct regression modeling of the effects of covariates on the CIF competing risks was utilized to detect the predictors of relapse. The variables used for this purpose were selected based on a univariate analysis and review of the literature. To determine the cut‐off used for our patients, we used the median of them. If the median threshold was not suitable for significance, the threshold suggested by other studies was used.

## 3 Results

**Figure 1:**
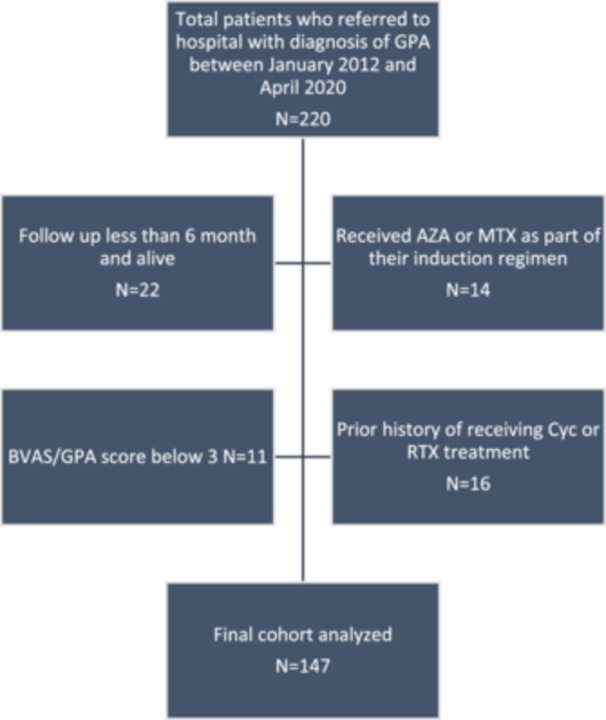
Final study cohort based on eligibility criteria.

During the study, 220 patients were referred to our tertiary hospital and diagnosed with GPA based on established diagnostic guidelines. Patients with a follow‐up duration of less than 6 months (*n* = 22), patients with prior history of receiving Cyc or RTX treatment (*n* = 16), patients who received AZA or MTX as part of their induction regimen (*n* = 14) and patients with a BVAS/GPA score below 3 (*n* = 11) were excluded (Figure 1). As a result, 147 patients met the eligibility criteria and are included in the present study. Supporting Information S1: Table S1 compares the baseline patient characteristics among initial cohort of patients (*n* = 220) versus final included patients (*n* = 147). Overall, the proportion of renal involvement (58.5% vs. 46.8%) and proportion of patients with severe disease (75.5% vs. 64%) was higher among final cohort. Age, gender, all other organ involvements, laboratory findings, treatments and follow‐up period were comparable among initial and final cohorts.

The males comprised 54.4% (*n* = 80), and the median age of patients was 45 (IQR: 34–61). Most patients (76.8%, *n* = 113) had a positive ANCA including PR3+ in 94 (63.9%) and MPO+ in 21 (14.2%). The median follow up time was 18 months (IQR: 6–38). All patients presented with ear, nose or throat (ENT) symptoms, 83% (*n* = 122) had lower respiratory system symptoms at presentation and 58.5% (*n* = 85) presented with renal symptoms. The median BVAS score of patients was 12 (IQR: 8–21) and 75.5% (*n* = 111) had severe disease based on BVAS/GPA classification. The most common laboratory finding was a high c‐reactive protein in 69.3% (*n* = 102) patients. Most patients received cyclophosphamide as induction therapy (82.3%, *n* = 121). All patients who received rituximab as their induction therapy were admitted after 2018. The summary of baseline patient characteristics is presented in Supporting Information S1: Table S1.

### 3.1 Survival Analysis, Cause of Death and Predictive Factors

**Figure 2:**
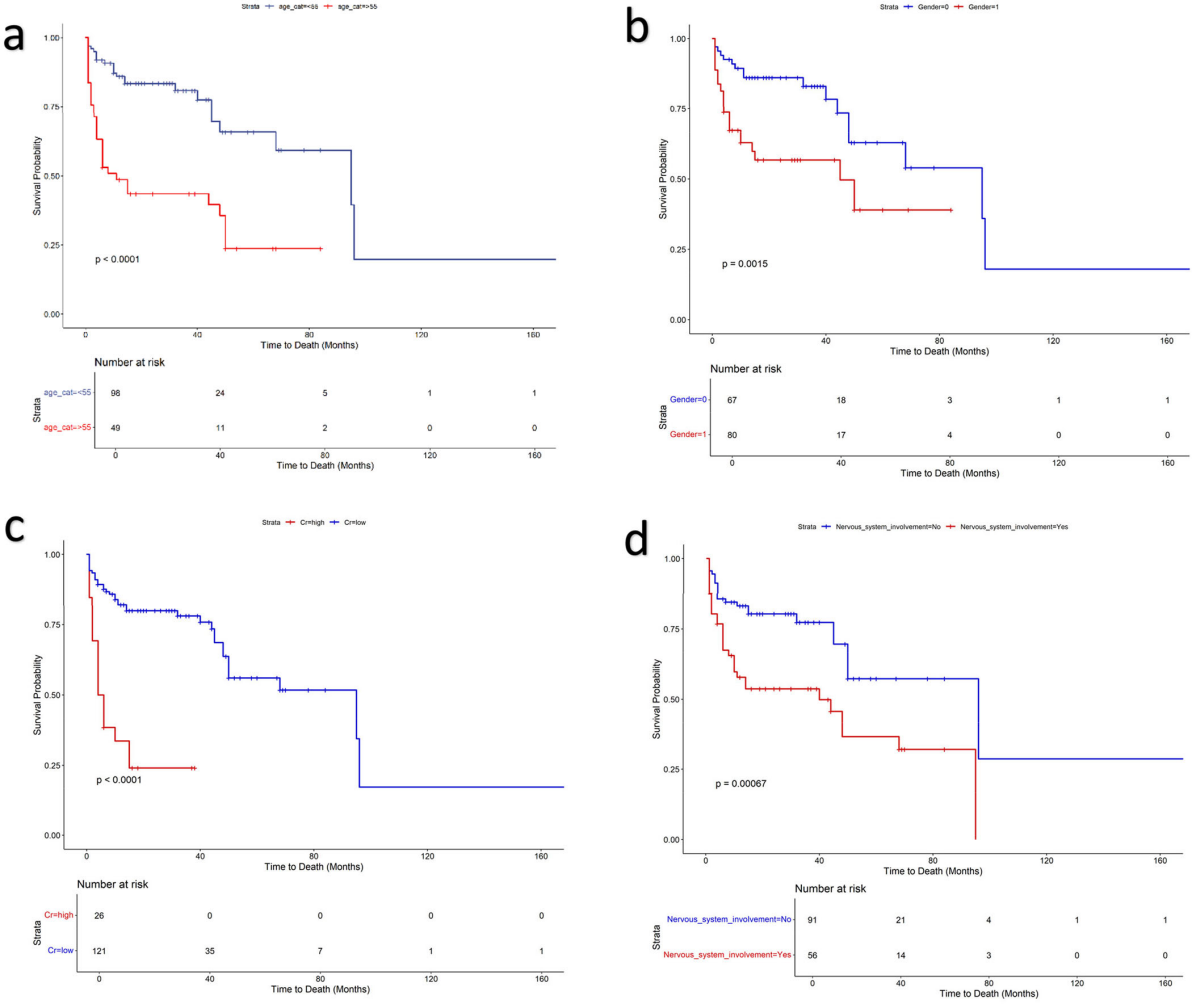
Kaplan‐Meier curve for significant predicting factors of mortality. The figure shows higher mortality in patients ≥ 55 years (a), male gender (b), rise of creatinine (c) and nervous system involvement (d).

We explored midterm survival (median follow up = 18 m) and prognostic factors of the enrolled patients. Figure 2 shows the survival of the enrolled patients in a median follow‐up of 18 months (IQR: 6–38 m). Overall, 55 deaths occurred leading to a survival rate of 62.5%. The leading causes of death were cardiovascular failure (38.2%, *n* = 21), followed by vasculitis (32.7%, *n* = 18), infection (21.8%, *n* = 12) malignancy (5.5%, *n* = 3), and GI bleeding (1.8%, *n* = 1). Table 1 compares the characteristics of patients who survived and those who died during the study period. Thos who died were older (median age: 57 vs. 36.5 years, *p* < 0.01), and had more severe disease at baseline (68% vs. 87%, *p* = 0.01). Gender was also a risk factor for death with males experiencing more deaths than women (47.5% in males 25.4% in females, *p* < 0.01). The organ involvement was similar among groups except nervous system involvement which was higher among fatalities (56.3% vs. 27.1%, *p* < 0.001). Elevated inflammatory factors at time of diagnosis including ESR+ and CRP + were also more prevalent among patients who died (CRP + : 85.4% vs. 59.7%, *p* < 0.01 and ESR + : 80% vs. 48.9%, *p* < 0.001). Rise of creatinine was also a risk factor (34.5% vs. 7.6%, *p* < 0.001). The induction treatment was also significantly different among cohort (*p* < 0.001). Among 26 patients who received rituximab, only one death occurred (3.8%) while among 121 patients who received cyclophosphamide 54 deaths occurred (44.6%). However, both groups had similar lag of diagnosis and ANCA status. Our Cox model found four independent factors for death after adjusting for other factors. The independent risk factors for mortality were: Age > 55 years with a HR of 2.40 (95% CI [1.24–4.61]), male gender with a HR of 2.23 (95% CI [1.17–4.25]), rise of creatinine > 1.3 mg/dL at presentation with a HR of 2.48 (95% CI [1.22–5.05]) and nervous system involvement with a HR of 2.16 (95% CI [1.20–3.89]). The final model had a concordance level of 0.79 and a *p* value of < 0.001. The final model for mortality is shown in Table 2. The Kaplan‐Meier curves for significant factors of mortality are shown in Figure 2.

**Table 1:** General characteristics of patients who survived and died during the follow up.

Group	Patients who survived at last follow up	Patients who died at last follow‐up	*p* value for difference between groups
	*N* = 92	*N* = 55	
Median Age, year (IQR)	36.5 (31–51)	57 (44–68)	< 0.001
Male/female, *n*	42/50	38/17	< 0.01
Lag of diagnosis, median (IQR) in months	12 (0‐24)	12 (0‐36)	0.6
ENT involvement, *n* (%)	92 (100)	55 (100)	—
Renal involvement, *n* (%)	50 (54.3)	36 (65.4)	0.2
Pulmonary involvement, *n* (%)	75 (81.5)	47 (85.4)	0.6
Mucous membranes/eyes, *n* (%)	34 (36.9)	26 (47.2)	0.2
Dermatologic involvement, *n* (%)	19 (20.6)	9 (16.3)	0.6
General involvement, *n* (%)	32 (34.7)	20 (36.3)	0.9
Cardiovascular involvement, *n* (%)	3 (3.2)	5 (9.0)	0.2
Nervous involvement, *n* (%)	25 (27.1)	31(56.3)	< 0.001
BVAS score, median (IQR)	12 (8‐20)	15 (8–24.5)	0.05
Severe disease based on BVAS/GPA	63 (68.4)	48 (87.2)	0.01
PR3‐ANCA +	59 (64.1)	35 (63.6)	1
MPO‐ANCA +	14 (15.2)	7 (12.7)	0.8
ANCA +	72 (78.2)	41 (74.5)	0.7
ANCA −	20 (21.7)	14 (25.4)	0.7
CRP ≥ 10	55 (59.7)	47 (85.4)	< 0.01
ESR ≥ 30 mm/h	45 (48.9)	44 (80)	< 0.001
Cr > 1.3 mg/dL	7 (7.6)	19 (34.5)	< 0.001
Leukocytosis (WBC > 11000)	34 (36.9)	28 (50.9)	0.1
Anemia (Hb < 12.5 mg/dL)	46 (50)	36 (65.4)	0.09
Induction with RTX	25 (27.2)	1 (1.8)	< 0.001
Induction with CyC	67 (72.8)	54 (98.2)	< 0.001

**Table 2:** Cox proportional hazard model for independent variables affecting mortality.

Variable	Hazard ratio (HR)	95% CI for HR	*p* value
Age ≥ 55 versus < 55	2.40	1.24–4.61	*< *0.001
Male versus Female	2.23	1.17–4.25	*< *0.001
rise of creatinine > 1.3 mg/dL versus normal creatinine	2.48	1.22–5.05	*< *0.01
Nervous system involvement versus no involvement	2.16	1.20–3.89	*< *0.01
CRP + versus CRP −	1.52	0.66–3.5	0.2
Rituximab versus cyclophosphamide	0.23	0.03–1.70	0.4
Severe disease versus limited disease	2.16	1.20–3.89	0.2

### 3.2 Determining the Predicting Factors for Relapse

In total, 179 relapses occurred among 117 patients (mean number of relapse per patient = 1.53) during the follow‐up period. There were 30 patients who had a relapse‐free survival in a median follow‐up of 19 months. Table 3 presents the baseline, laboratory and treatment features of patients who relapsed and did not relapse. Patients who remained relapse‐free during follow‐up, compared to those who relapsed or passed away, were more likely to have received rituximab. They also exhibited lower frequencies of mucus/eye involvement, less general manifestations, lower baseline BVAS scores, reduced anemia rates, and less pronounced elevations in inflammatory markers (Table 3). Our competitive‐risk analysis showed that the type of treatment (Cyclophosphamide vs. Rituximab) was an independent risk for relapse with (Gray's test *p *< 0.001) (Figure S1). However, after running direct regression modeling, the type of treatment was not found as an independent predictor for relapse. Table 4 summarizes the final model for risk factors of relapse. The final model included four independent factors for relapse including: Age > 55 with a relative risk (RR) of 2.50 (95% CI [1.20–2.51]), male gender with a RR of 2.17 (95% CI [1.10–4.28]), rise of creatinine at baseline with a RR of 2.24 (95% CI [1.15–4.35]) and nervous system involvement with a RR of 2.18 (95% CI [1.21–3.96]).

**Table 3:** General characteristics of patients who relapsed and did not relapse during the follow‐up.

Group	Alive patients who did not relapse (*n* = 30)	Patients who relapsed (*n* = 117)	*p* value between groups
Median follow‐up time, month (IQR)	19 (9–29)	18 (6–40)	0.4
Male/female, *n*	13/17	67/40	0.2
Age, median, IQR	45 (33–57)	45 (35–62)	0.9
Induction with rituximab	16 (53)	10 (8)	< 0.001
Induction with cyclophosphamide	14 (47)	107 (92)	< 0.001
ENT involvement, *n* (%)	30 (100)	117 (100)	—
Renal involvement, *n* (%)	16 (53)	70 (60)	0.6
Pulmonary involvement, *n* (%)	23 (77)	99 (84.6)	0.4
Mucous membranes/eyes, *n* (%)	5 (20)	55 (47)	< 0.001
Dermatologic involvement, *n* (%)	2 (7)	26 (22)	0.09
General involvement, *n* (%)	4 (13)	48 (41)	< 0.01
Cardiovascular involvement, *n* (%)	0 (0)	8 (7)	0.3
Nervous involvement, *n* (%)	10 (33)	46 (39)	0.7
BVAS score, median (IQR)	10 (7–15)	14 (8–22)	< 0.01
Severe disease based on BVAS/GPA	19 (63)	92 (78)	0.1
PR3‐ANCA +	16 (53)	78 (67)	0.2
MPO‐ANCA +	6 (20)	15 (13)	0.4
ANCA −	8 (27)	26 (23)	0.7
CRP ≥ 10	15 (50)	87 (74)	0.01
ESR ≥ 30 mm/h	9 (30)	80 (68)	< 0.001
Cr > 1.3 mg/dL	3 (10)	23 (19)	0.3
Leukocytosis (WBC > 11000)	9 (30)	53 (45)	0.1
Anemia (Hb < 12.5 mg/dL)	11 (36)	71 (60)	0.03

**Table 4:** Direct regression modeling in a competitive risk setting for independent variables affecting relapse‐free survival.

Variable	Relative risk (RR)	95% CI for RR	*p* value
Age ≥ 55 versus < 55	2.50	1.2–5.1	*< *0.01
Male versus Female	2.17	1.10–4.28	0.02
Rise of creatinine > 1.3 mg/dL versus normal creatinine	2.24	1.15–4.35	0.01
Nervous system involvement versus no involvement	2.18	1.21–3.96	< 0.01
Induction treatment with cyclophosphamide versus Rituximab	5.08	0.63–40.7	0.1
General system involvement	0.97	0.55–1.71	0.9
Initial BVAS score, > 21 versus < 21	1.13	0.68–1.87	0.6
CRP + versus CRP −	1.40	0.52–3.80	0.5
Anemia at presentation	1.47	0.82–2.65	0.1
ESR + versus ESR −	0.81	0.28–2.34	0.7
Mucus/eye involvement	1.12	0.62–2.02	0.6

## 4 Discussion

The aim of this study was to determine the effect of baseline clinical manifestations, laboratory findings, and disease severity on the prognosis of GPA patients. We found that Some demographic, clinical, and laboratory factors that can be assessed when diagnosing patients and be helpful in predicting a patient's relapse and mortality. Awareness of this information can lead to better understanding of subgroups of patients with more vulnerability and as a result, earlier identification and treatment of these patients.

In this study, we included consecutive patients who were referred to our tertiary hospital in an 8 year period. This criterion led to the admission of 220 patients in initial cohort, which is a relatively large sample size compared to similar studies [30, 31, 32]. One of the reasons for this large sample size could be related to the unique features of our hospital. Our hospital is a referral center from all over the country for patients with ENT symptoms, and since most GPA patients have ENT symptoms at the beginning of the disease, it is expected that the number of patients identified be remarkable.

In this study, the effect of the involvement of different organs in two prognostic criteria including relapse rate and mortality was investigated. Our results highlight that male gender, older age, the presence of nervous system involvement, and renal dysfunction (evidenced by increased creatinine) are critical prognostic factors. These patient demographics and clinical presentations are not only linked to an elevated risk of relapse but also predict a higher mortality rate. However other clinical symptoms had no effect on the relapse rate or mortality. It's noteworthy, and perhaps initially surprising, that these same variables predict both relapse and mortality. However, interpreting this shared prediction requires careful consideration, particularly within the framework of a competing risk analysis. Given the relatively high mortality rate observed in our patient cohort (37.5% in a median 18 months follow up), it's possible that the strong influence of mortality as a competing event could impact how certain factors appear to predict relapse. In essence, patients with these adverse characteristics might be at such a high risk of mortality that their likelihood of experiencing relapse as a distinct event could be intertwined with, or even curtailed by, the competing risk of death. Our analysis accounts for this complexity, providing a more robust understanding of these interconnected risks.

In 2005, Hogan et al. [33] examined the factors influencing relapse and found that lung and upper respiratory tract involvement were each associated with a 1.7‐fold increased risk of relapse, while kidney and skin involvement were not statistically significant. In contrast, Koldingsnes et al. [12] reported only those with cardiovascular involvement at the beginning of the disease have a higher risk of relapse. In the present study, upper respiratory tract involvement could not be assessed because all patients had these symptoms. However, pulmonary or cardiovascular involvement in the present study was not associated with an increase in relapse risk or mortality. It seems that the characteristics of the patients studied influence these results. A recent meta‐analysis has proposed pulmonary, cardiovascular, upper respiratory, and gastrointestinal involvement as associated organs with higher relapse risk [34]. Regarding the importance of renal function in the prognosis of AAV, Sanchez‐Alamo et al, reported that even mild forms of kidney involvement is a negative prognostic factor [35]. Regarding the effect of nervous system involvement, we have previously shown that neurologic symptoms should be considered as red flag, since their presentation is associated with higher morbidity and mortality [36]. While our work, and existing literature, clearly highlights the impact of neuropathy on patient mortality, its specific effect on the risk of relapse warrants more dedicated investigation. The interplay between neurological involvement and the likelihood of disease relapse is a complex area that could offer crucial insights for long‐term patient management. Therefore, we strongly recommend that future studies specifically explore and characterize the influence of neuropathy on relapse risk.

Regarding the mortality rate, we can refer to the study of Flossmann et al. [37]. They reported 25% death is a median follow up of 5.2 years in their cohort of AAV patients, which is significantly lower than our observed mortality rate of 37.5% in a median follow up of 1.5 years. The higher observed mortality rate in our study compared to Flossmann et al.'s cohort could be attributed to several factors. First, the delayed time of diagnosis, with a median lag of 12 months, likely resulted in patients presenting with more advanced disease stages and complications, negatively affecting survival outcomes. Additionally, our study exclusively focused on GPA patients, whereas Flossmann et al. examined a broader cohort of AAV patients, which may lead to different survival rates. Another significant factor is the study setting; conducting our research in a single‐center within a developing country introduces disparities in healthcare access, treatment availability, and overall management, compared to studies performed in developed regions. Differences in treatment protocols, the availability of advanced therapeutic options, and healthcare infrastructure may further contribute to the observed discrepancy.

In 2017, Haris et al. [14] stated that paying attention to simple metrics that can be measured at diagnosis, helps to predict a patient's relapse and mortality. Accordingly, they showed that middle‐aged individuals with higher BVAS were at higher risk of relapse and mortality. In the present study, the median BVAS score of patients was 11, which had no effect on estimating relapse and prognosis, and the BVAS score of 21 suggested by Haris et. al was also insignificant. Yoo et al. [15] also pointed to the role of BVAS in the prognosis of the disease and found similar results. In 2004, Bligny et al. [13] examined the factors affecting the mortality rate of GPA patients and stated that in the univariate analysis of variables over the age of 52, the absence of ENT involvement, high creatinine, and anemia are associated with a worse prognosis. However, in the final analysis, only ages over 52 years and no involvement of the ear, nose, and throat remained. Similarly, in the present study, age over 55 years was considered a determining factor in the death rate of patients. Takala et al. also stated that patients with kidney involvement and older age at diagnosis were more likely to die which was in line to our study findings [38].

Although this study is a large scale study with a specific subgroup of AAV (only GPA), it has many limitations. First, this was a retrospective study with an inherent risk of selection bias. We only had short‐term follow up data and the data on maintenance therapy was not complete for all patients which hinders our ability to assess long‐term outcomes and the effectiveness of different treatment strategies. Additionally, the study was conducted in a single‐center setting, which may limit the generalizability of the findings to broader populations with diverse healthcare systems and treatment approaches. The delayed diagnosis observed in this cohort likely influenced disease severity at presentation, further impacting survival and relapse rates, however this is in line with the real‐world setting in a developing country. Furthermore, variations in treatment protocols and access to advanced therapies over the study period of 8 years may have contributed to differences in patient outcomes over time. Another limitation of this study is the exclusion of patients who received AZA or MTX as induction treatment. Although this exclusion applied to a small number of patients (*n* = 14) and is unlikely to have significantly impacted the overall findings of this manuscript, we acknowledge its potential relevance for broader generalizability. Despite the current recommended induction regimens primarily being Cyclophosphamide or Rituximab, we recognize that in real‐world clinical practice, alternative agents like AZA or MTX are sometimes utilized. Therefore, for future studies, particularly those focused on real‐world outcomes, we strongly suggest including patients treated with these alternative induction regimens. This would provide a more comprehensive understanding of treatment effectiveness and patient outcomes across the full spectrum of clinical practice.

## 5 Conclusion

GPA is a highly relapsing disease that can lead to death if left untreated. Some demographic, clinical, and laboratory factors that can be assessed when diagnosing patients can be helpful in predicting a patient's relapse and mortality. Male patients aged 55 and older who experience nervous system complications or elevated creatinine levels face a heightened risk of relapse and mortality. Due to these increased risks, a more intensive treatment approach is advised for this group.

## Author Contributions

**Farzaneh Kianifar:** supervision, conceptualization, writing – review and editing, writing – original draft, data curation. **Mohammad Eslami:** conceptualization, supervision, writing – review and editing, writing – original draft, data curation. **Soheil Tavakolpour:** conceptualization, writing – original draft, formal analysis, visualization. **Noushin Afshar Moghaddam:** conceptualization, writing – original draft, formal analysis, visualization. **Samira Alesaeidi:** conceptualization, supervision, writing – original draft, writing – review and editing. **Seyed Behnam Jazayeri:** conceptualization, writing – review and editing.

## Ethics Statement

The study obtained approval from the Ethics Advisory Committee of the Research Vice‐Chancellor at Tehran University of Medical Sciences in Iran (Approval ID: IR.TUMS.AMIRALAM.REC.1399.036) before its commencement. The research was conducted in accordance with good clinical practice guidelines, and all participants provided written informed consent to participate in the study.

## Conflicts of Interest

The authors declare no conflicts of interest.

## Transparency Statement

The lead author Seyed Behnam Jazayeri affirms that this manuscript is an honest, accurate, and transparent account of the study being reported; that no important aspects of the study have been omitted; and that any discrepancies from the study as planned (and, if relevant, registered) have been explained.

## Supporting information


**Figure S1:** Competing risk analysis for relapses considering the induction treatment. **Table S1**: General characteristics of patients.

## Data Availability

The data regarding this manuscript is made available upon reasonable request from the corresponding author.
