# Supplementary material for: The Prognosis of Granulomatosis With Polyangiitis: The Risk of Relapse and Mortality Based on Baseline Clinical Manifestations, Laboratory Findings, and Disease Severity: A Retrospective Cohort Study
Source: Health Sci Rep. 2025 Sep 14;8(9):e71239. doi: 10.1002/hsr2.71239 (PMC12434175; doi:10.1002/hsr2.71239)
Supplement: Supplementary file 1 — Figure S1: Competing risk analysis for relapses considering the induction treatment. Table S1: General characteristics of patients. [file HSR2-8-e71239-s001.docx]

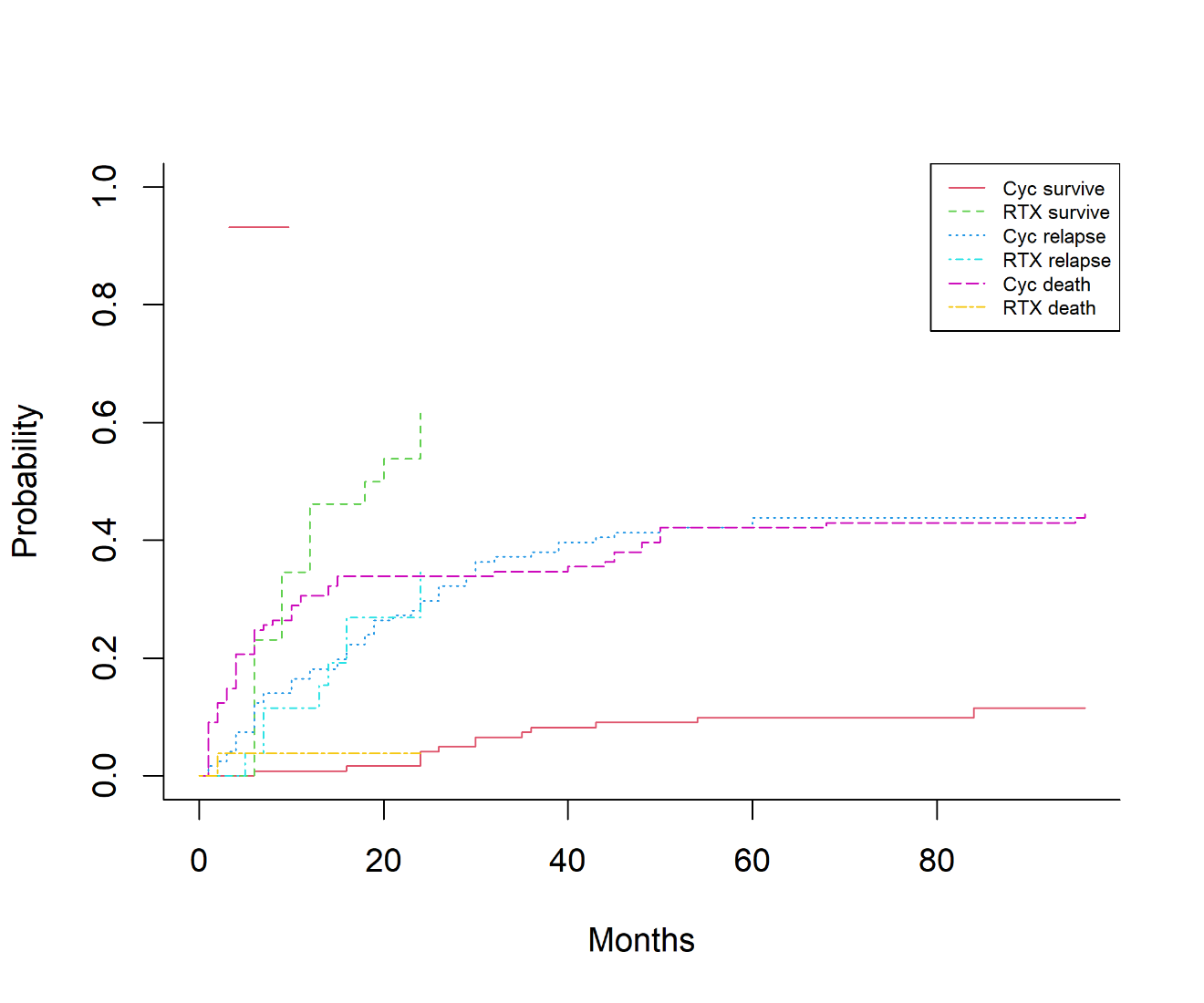


**Figure S1. Competing risk analysis for relapses considering the induction treatment**

**Table S1**: General characteristics of patients

| Group | Total cohort  (n=220) | Included in analysis  (n=147) | P value for difference between groups |
| --- | --- | --- | --- |
| Median follow-up time, month (IQR) | 19 (4-24) | 18 (6-38) | 0.39 |
| Median Age, year (IQR) | 43 (32-57) | 45 (34-61) | 0.27 |
| Male/female, n | 112/108 | 80/67 | 0.57 |
| ENT involvement, n (%) | (99.5) 219 | 147 (100) | - |
| Renal involvement, n (%) | (46.8) 103 | 86 (58.5) | 0.03 |
| Pulmonary involvement, n (%) | (76.8) 169 | 122 (83) | 0.19 |
| Mucous membranes/eyes, n (%) | (38.1) 84 | 60 (40.8) | 0.9 |
| Dermatologic involvement, n (%) | (13.6) 30 | 28 (19) | 0.21 |
| General involvement, n (%) | (33.1) 73 | 52 (35.3) | 0.74 |
| Cardiovascular involvement, n (%) | (4.5) 10 | 8 (5.4) | 0.88 |
| Nervous involvement, n (%) | (34.5) 76 | 56 (38) | 0.55 |
| BVAS score, Median (IQR) | 13.5 (3-40) | 12 (8-21) | 0.07 |
| Severe disease based on BVAS/GPA | 141 (64) | 111 (75.5) | 0.02 |
| PR3-ANCA + | (63.3) 138 | 94 (63.9) | 0.89 |
| MPO-ANCA + | (13.3) 29 | 21 (14.2) | 0.88 |
| ANCA + | (75.2) 164 | 113 (76.8) | 0.70 |
| ANCA - | (24.8) 54 | 34 (23.2) | 0.85 |
| CRP≥ 10 | (62.8) 137 | 102 (69.3) | 0.19 |
| ESR≥ 30 mm/h | (55.9) 122 | 89 (60.5) | 0.39 |
| Cr>1.3 mg/dL | (17.4) 38 | 26 (17.6) | 1 |
| Leukocytosis (WBC>11000) | (34.4) 75 | 62 (42.1) | 0.14 |
| Anemia (Hb<12.5 g/dL) | (52.7) 115 | 82 (55.8) | 0.57 |
| Induction with RTX | 26 (11.8) | 26 (17.7) | 0.1 |
| Induction with Cyc | 180 (81.8) | 121 (82.3) | 1 |
